# Are we there yet? Initial targeting of the Male-Specific Lethal and Polycomb group chromatin complexes in *Drosophila*

**DOI:** 10.1098/rsob.140006

**Published:** 2014-03-26

**Authors:** Kyle A. McElroy, Hyuckjoon Kang, Mitzi I. Kuroda

**Affiliations:** 1 Department of Molecular and Cellular Biology, Harvard University, Cambridge, MA 02138, USA; 2 Department of Medicine, Division of Genetics, Brigham and Women's Hospital, Boston, MA 02115, USA; 3 Department of Genetics, Harvard Medical School, Boston, MA 02115, USA

**Keywords:** chromatin binding, *Drosophila*, Male-Specific Lethal complex, Polycomb group targeting

## Abstract

Chromatin-binding proteins must navigate the complex nuclear milieu to find their sites of action, and a constellation of protein factors and other properties are likely to influence targeting specificity. Despite considerable progress, the precise rules by which binding specificity is achieved have remained elusive. Here, we consider early targeting events for two groups of chromatin-binding complexes in *Drosophila*: the Male-Specific Lethal (MSL) and the Polycomb group (PcG) complexes. These two serve as models for understanding targeting, because they have been extensively studied and play vital roles in *Drosophila*, and their targets have been documented at high resolution. Furthermore, the proteins and biochemical properties of both complexes are largely conserved in multicellular organisms, including humans. While the MSL complex increases gene expression and PcG members repress genes, the two groups share many similarities such as the ability to modify their chromatin environment to create active or repressive domains, respectively. With legacies of in-depth genetic, biochemical and now genomic approaches, the MSL and PcG complexes will continue to provide tractable systems for understanding the recruitment of multiprotein chromatin complexes to their target loci.

## 2. Introduction

The primary unit of chromatin is the nucleosome, consisting of approximately 147 base pairs of DNA wrapped around a histone octamer. Genetic information is encoded at the DNA level, but most interpretation of genetic information occurs at the protein level. In addition to histones, numerous non-histone proteins interact with chromatin and DNA to bring about the proper, cell-specific interpretation of the genome.

**Figure 1.**
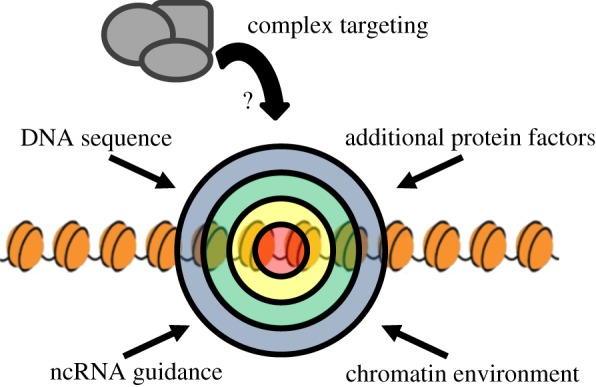
Potential factors influencing the selection of chromatin-binding sites. Numerous characteristics may influence the selection of binding sites in the genome. DNA primary sequence and sequence composition, the local chromatin environment and long-range chromatin conformation, protein–protein interactions and non-coding RNA guidance may act synergistically for proper targeting of chromatin complexes to their sites of action.

One major question in chromatin biology is how protein players find their proper sites of action. In genomes ranging in sizes from megabases to gigabases of DNA, transcription factors, which recognize their sites in a DNA sequence-specific manner, are only found localized to a fraction of their numerous consensus sequences throughout the genome. It is no wonder, then, that complete understanding of the potentially more complicated phenomenon of chromatin targeting has remained elusive (figure 1). Here, we focus on two groups of chromatin-bound factors in *Drosophila*: the male-specific lethal (MSL) complex and the Polycomb group (PcG). These two groups can be considered as models for addressing how chromatin factors are targeted, as their sites of action have been documented at high resolution.

**Figure 2.**
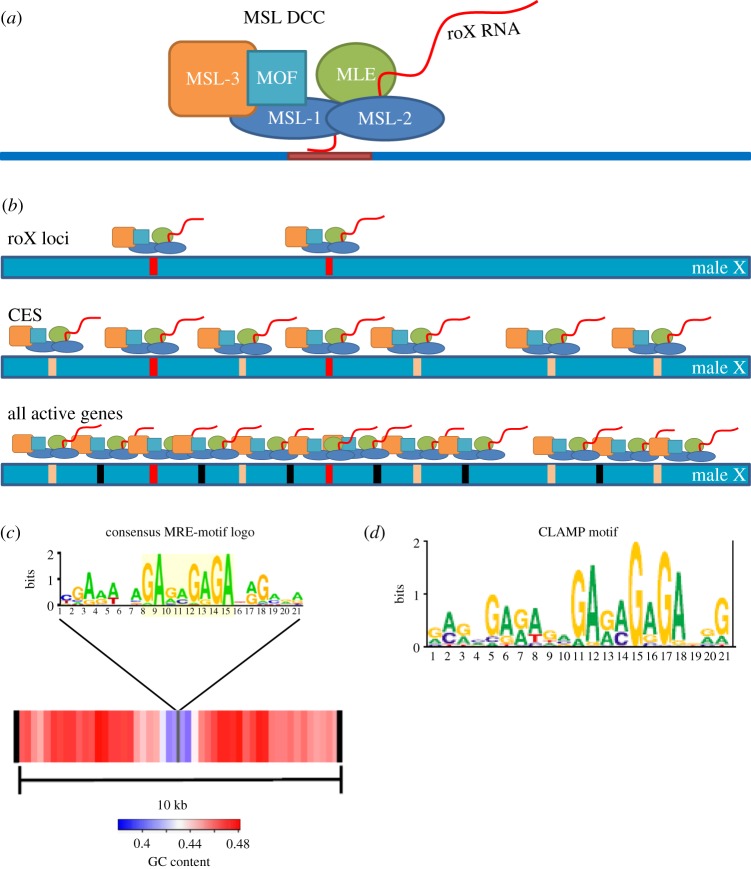
The MSL spreading model, the MRE and CLAMP. (*a*) The Male-Specific Lethal dosage compensation complex consists of five core protein members and one of two non-coding RNAs. Male-specific lethal (MSL) 1, 2 and 3, Males absent on the first (MOF) and Maleless (MLE) are thought to initially assemble cotranscriptionally with one of the two *roX* (*RNA on the X*) RNAs. The MOF subunit acetylates histone 4 at lysine 16 (H4K16Ac). Adapted from Gelbart & Kuroda [1]. (*b*) The MSL complex is proposed to spread to most active genes on the male X chromosome in a stepwise model. First, the complex is assembled cotranscriptionally at the *roX* loci (red boxes), and then to approximately 250 chromatin entry sites (CESs) along the X in a sequence-dependent manner (peach boxes). Finally, the complex spreads from the CESs to most active genes in a sequence-independent manner (black boxes). Adapted from Gelbart & Kuroda [1]. (*c*) Analysis of the MSL recognition elements (MREs) within CESs revealed a degenerate GA-rich sequence that is required for MSL binding when tested in a transgenic context. Additionally, the regions immediately surrounding the CESs have low GC-content generally, whereas the 10 kb flanking regions are generally GC rich. From Alekseyenko *et al.* [2,3], reproduced with permission. (*d*) The direct DNA-binding sequence motif identified for CLAMP is very similar to the observed MRE sequence. From Soruco *et al.* [4].

The MSL complex regulates dosage compensation in *Drosophila*. Dosage compensation is the means by which X chromosome gene expression is adjusted to balance gene expression from the autosomes. In flies, this is achieved by hypertranscription of the active genes on the single male X chromosome. The MSL complex is made up of five core proteins (figure 2*a* and table 1) and two redundant non-coding RNAs (roX1 and roX2). The loss of any protein component or both RNAs leads to male lethality. Downstream of the sex determination cascade, the MSL complex is assembled in males only and targeted exclusively to active genes on the X chromosome. Recruitment to the male X appears to occur in at least two steps (figure 2*b*). The first step involves initial targeting to several hundred chromatin entry sites (CESs; also called high-affinity sites) carrying a degenerate sequence motif. Two prominent CESs are the *roX* RNA genes that produce the ncRNA components of the complex. The second targeting step involves sequence-independent spreading in *cis* to most active genes. It is for this second step that roX RNAs seem most critical (reviewed in reference [1]). Evidence for this ‘nucleate and spread’ model comes from experiments in which the MSL complex becomes targeted to active genes flanking the ectopic insertion of *roX* RNA transgenes on autosomes [51,52]. Therefore, the initial targeting of the complex to X chromosome entry sites is critical for its specificity. However, understanding the mechanism for the selection of the initial CES has been challenging, because the associated sequence motif is enriched less than twofold on the X chromosome versus the autosomes.

**Table 1.** Genetically defined members of the MSL complex and the PcG group. The genetically identified members of the MSL complex and the PcG group are listed as found in purified complexes [5–12]. Known RNA/DNA/chromatin interaction domains are listed as well as additional domains not typically observed to have this function. Structural studies have informed the understanding of molecular mechanisms. Relevant *Drosophila* structural data are provided with protein data bank (PDB) identifiers. Escl, Extra sex combs-like; Pcl, Polycomb-like; Sxc (Ogt), Super sex combs (*O*-glycosyltransferase); Crm, Cramped.

protein	complex	domains associated with RNA/DNA/chromatin interaction	other domains	relevant *Drosophila* structural data—protein data bank (PDB) IDs	domain and structural references
MSL1	MSL [12]		coiled-coil, PEHE		[13,14]
MSL2	MSL	CXC	RING	CXC—2LUA	[14–17]
MSL3	MSL	chromodomain	MRG	chromodomain—3M9Q	[18–20]
MOF	MSL	chromodomain, Zn finger (C2HC), HAT		chromodomain—2BUD	[21,22]
MLE	MSL	RB1, RB2, ATPase/helicase, Gly rich			[23,24]
Pc	PRC1 [5]	chromodomain		chromodomain—1PFB, 1PDQ	[25–27]
Ph	PRC1	Zn finger (FCS)	SAM	SAM—1PK1, 1KW4	[28,29]
Psc	PRC1/dRAF [9]		RING		[30]
Su(z)2			RING		[30]
dRing	PRC1/dRAF		RING		[31–33]
Scm	PRC1	Zn finger (FCS)	MBT, SAM	SAM—1PK1, 1PK3; MBT—2R57, 2R58, 2R5A, 2R5M	[28,34,35]
E(z)	PRC2 [6–8]	SANT, SET	CXC		[36,37]
Esc	PRC2		WD40		[38]
Escl			WD40		[39]
Su(z)12	PRC2	Zn finger (C2H2)	VEFS-box	Su(z)12/Nurf55 interaction—2YB8	[40,41]
Pcl	PRC2		Zn fingers (PHD), Tudor	Tudor—2XK0	[42,43]
Pho	PhoRC [11]	Zn finger (C2H2)		Pho/Sfmbt interaction—4C5E, 4C5G, 4C5H	[44,45]
Phol		Zn finger (C2H2)			[46]
dSfmbt	PhoRC	Zn finger (FCS)	MBT, SAM	MBT—3H6Z; Pho/Sfmbt interaction—4C5E, 4C5G, 4C5H	[44,47]
Asx	PR-DUB [10]		Zn finger (PHD)		[48]
Calypso	PR-DUB		Peptidase C12		[10]
Sxc (Ogt)			TPR		[49]
Crm		SANT			[50]

**Figure 3.**
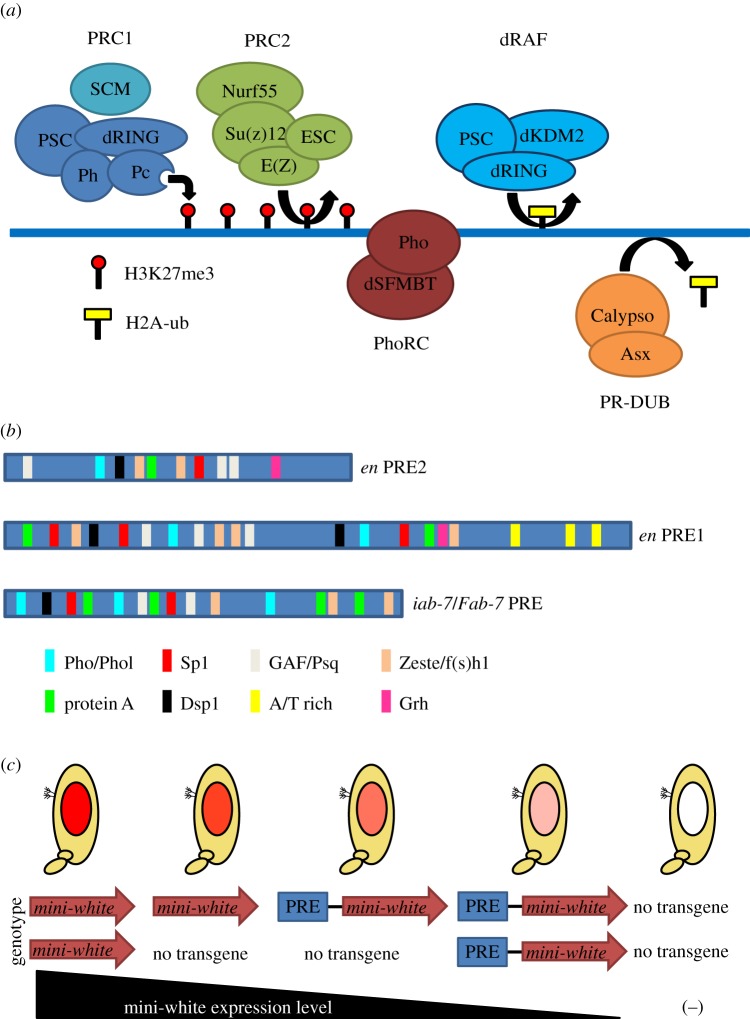
PcG complexes, PRE architecture and pairing-sensitive silencing (PSS). (*a*) Multiple polycomb group complexes have been characterized: Polycomb Repressive Complex 1 and 2 (PRC1, PRC2), Pho-repressive complex (PhoRC), dRING-associated factors (dRAF) and Polycomb repressive deubiquitinase (PR-DUB). E(z) catalyses trimethylation of histone 3 on lysine 27 (H3K27me3). The chromodomain of Pc is known to recognize this mark. Pho (and the related Phol) are the only PcG proteins to have characterized sequence-specific binding. dRING (also called Sce, Sex combs extra) catalyses H2A ubiquitination in the context of the dRAF complex, but not in the context of PRC1. The PR-DUB complex removes this H2A ubiquitination. The reason for ubiquitination cycling on H2A is not fully understood. Adapted from Schwartz & Pirrotta [53]. (*b*) The *engrailed* PREs and the *iab-7/Fab-7* PRE are schematized with identified consensus sequences for various DNA-binding factors. Despite the large number of potential interactors, no single motif is sufficient to predict PREs. Adapted from Brown & Kassis [54]. (*c*) PSS is a phenomenon in which expression of a homozygous transgene that contains a PRE is less than in the heterozygous case, suggesting that homologue pairing enhances silencing mediated by the PRE. In comparison, homozygosity for transgenes lacking PREs generally leads to higher expression levels. The gradation of expression is schematized for a mini-white transgene in a *white^–/−^* genetic background. The *white* gene is responsible for red eye colour in flies.

The PcG functions as a set of repressors that maintain the transcriptional inactivation of developmentally silenced genes. Originally identified as critical for the maintenance of the parasegment-specific pattern of Hox gene expression, subsequent analysis has identified hundreds of PcG targets beyond the Hox gene clusters. The PcG is made up of approximately 20 proteins (figure 3*a* and table 1), which form several multiprotein complexes that possess slightly different genomic binding patterns and have differential biochemical activities. At individual target genes, Polycomb Response Elements (PREs) have been identified that can function in ectopic chromatin contexts, but these lack a strong consensus motif. A classical model for the targeting of PcG complexes is that they recognize PREs in silenced domains that were previously established by repressive, spatially restricted transcription factors. Once PcG complexes are initially targeted to PREs, they can be stably maintained at these loci even after the original silencing factors are no longer expressed. Like the MSL complex, the PcG may also have a spreading mechanism, as silenced regions can form large PcG-associated domains. The creation of these domains, which can differ from cell type to cell type, is not understood.

In this review, we focus on the initial steps of recruitment of these complexes, which are likely to be mechanistically separable from later maintenance phases. For the MSL complex, the CESs comprise the set of sites that are initially targeted. For the PcG, PREs are generally considered ‘initial targets’. In the context of this review, PREs guide the PcG to lineage-specific target sites during embryonic development. Later phases of PcG-association probably form a self-perpetuating chromatin state, where complex retention at target sites is stable through the cell cycle, possibly by a modified ‘nucleate and spread’ mechanism from selectively retained sites [55–57].

## 3. Biochemical toolbox: DNA/chromatin recognition properties of the core complexes

In trying to understand the targeting of protein complexes to their sites of action throughout the genome, there are several factors to consider (figure 1). As a starting point, we catalogue the proteins of our model groups, especially taking note of their domain architecture relevant to DNA/chromatin interaction.

The MSL complex is targeted to the male X chromosome with virtually complete fidelity. It remains a mystery how this is accomplished with our current understanding of the members of the complex and their functions. An examination of the known domains present in the MSL proteins reveals that none is predicted to be a sequence-specific DNA-binding protein. Several, however, carry domains well characterized to interact with chromatin. Additionally, each member may confer unique biochemical activities to the whole.

There are two main enzymatic properties known to be required for MSL function. The first, RNA helicase activity, is conferred by Maleless (MLE) [23], which recent work suggests may be critical for roX RNA remodelling and complex assembly [58]. The second, histone acetyltransferase activity directed towards H4K16, is catalysed by Males absent on the first (MOF) [21,59], which is likely to be key to the increase in transcriptional activity of male X-linked genes. While these activities are both essential for MSL function, they appear to be dispensable for the initial targeting of the complex. More recently, the groups of Dou and Becker showed that MSL2 has E3 ubiquitin ligase activity *in vitro*. Wu *et al*. provided evidence that mammalian and fly MSL2 had activity in association with MSL1, leading to ubiquitination of H2B K34 (H2B K31 in flies) [60]. By contrast, Villa *et al*. found that *Drosophila* MSL2 ubiquitinates other MSL components, probably serving a stoichiometry-balancing role [61]. Whether either of these functions is essential for dosage compensation has not yet been reported.

Non-enzymatic domains that have known chromatin interaction capabilities are also present. MSL3 and MOF both contain chromodomains, which are found in various chromatin-modifying proteins and often interact with methylated histones. The MSL3 chromodomain is characterized to have H3K36me3 binding [52], H4K20me1 binding [18] and H4K20me2 binding [62]. The MSL3 chromodomain plays a role in the spread of MSL to all active genes, but, akin to the enzymatic activities above, is dispensable for initial targeting [63]. Unsurprisingly, MLE contains several RNA-interacting motifs, including a double-stranded RNA-binding domain, a DExH helicase domain and a C terminal glycine-rich region, which could be used for engaging chromatin via RNA [24,64].

Fauth *et al.* examined the DNA-binding capacity of the MSL1 and MSL2 proteins *in vitro*, using electrophoretic mobility shift assays with recombinant proteins, to demonstrate that the CXC domain in MSL2 exhibited non-specific affinity for DNA [15]. Recently, Straub and co-workers manipulated biochemical conditions prior to immunoprecipitation, in a novel approach to probe for high affinity protein–DNA interactions that might be captured within the nucleus. They used low-percentage formaldehyde to reduce the number of chemical cross-linking events and high-energy chromatin shearing to disrupt indirect protein–DNA interactions, ideally preserving only the most DNA–proximal interactions [65]. Using this method, MSL proteins are partially degraded, and surviving epitopes for MLE and MSL2 colocalize at CESs, suggesting that they may directly contact the initial targeting sites. This result is in contrast to previous genetic models, in which MSL1 and MSL2 function together at CESs [1]. In the high-energy-shearing experiments, MSL3 maintains its normal, broad localization. Perhaps because of its small size, MSL3 can survive the high-energy shearing the best, thus leaving its full pattern apparent. Cognate experiments using the ChIP-exo [66] or MNase ChIP techniques [67,68], which allow greater resolution without the potential loss of protein integrity from high-energy chromatin shearing, would be of great value to confirm the proposed high-resolution mapping of MSL2 and MLE specifically to CESs.

Similarly, the PcG contains many proteins that have chromatin-interacting domains, but few that are capable of binding DNA in a sequence-specific manner. A substantial number of the known PcG proteins have been identified as subunits of two main PcG complexes, Polycomb Repressive Complex 1 and 2 (PRC1 and PRC2). PRC1 complex is thought to act as a direct executor of target gene silencing through inhibition of chromatin remodelling and chromatin compaction [5]. Polycomb (Pc), Polyhomeotic (Ph), Posterior sex combs (Psc) and Sex comb extra (Sce, aka dRing) are the core subunits of PRC1 [31]. Suppressor of zeste 2 (Su(z)2), which is functionally redundant with Psc [69], and Sex comb on midleg (Scm) copurify with PRC1 at substoichiometric levels and are also categorized as PRC1 subunits. RNA interference (RNAi) knockdown experiments in tissue culture cells have suggested that Scm may be particularly important for targeting of PcG complexes at PREs [70].

The PRC2 complex is responsible for trimethylation of histone H3 lysine 27 (H3K27me3) [6–8,71], a mark of transcriptional repression which is known to be recognized by the chromodomain of Pc. PRC2 consists of Enhancer of zeste (E(z)) as its catalytic subunit, and Suppressor of zeste 12 (Su(z)12), Extra sex combs (Esc) and Nurf55 (aka Caf1) as non-catalytic subunits. Recent work suggests that the zinc-finger domain found in Su(z)12 may play a role in association with chromatin, as its deletion led to loss of viability in transgenic lines and loss of localization to PREs in cell culture [72]. It is yet unknown whether this is a direct interaction, or whether it is mediated by another accessory factor or factors.

In addition to PRC1 and PRC2, other PcG complexes have been identified in *Drosophila*. dRING-associated factors (dRAF) complex, which shares Psc and Sce/dRing with PRC1, contains the demethylase dKDM2, and is involved in H3K36me2 demethylation and H2A ubiquitylation [9]. Polycomb repressive deubiquitinase (PR-DUB), another PcG complex, consists of Additional sex combs (Asx) and the ubiquitin carboxy-terminal hydrolase Calypso, which specifically removes monoubiquitin from histone H2A [10]. The mutant phenotypes of these proteins demonstrate that the ubiquitination/de-ubiquitination cycle of H2A is important for PcG repression.

The Pho-repressive complex (PhoRC), consisting of the DNA-binding proteins Pleiohomeotic (Pho) or Pleiohometic-like (Phol) together with Scm-related gene containing four mbt domains (Sfmbt), is the only PcG complex shown to have sequence-specific DNA-binding activity [11,44]. Evidence for an interaction between PRC2 components and the DNA-binding proteins Pho and Phol, as well as the requirement of Pho in PRE binding of E(z), led to a model of hierarchical recruitment of PcG complexes [73]. In this model, Pho and Phol bind to PREs and recruit PRC2 complex to PREs through their interaction. Subsequently, E(z) methylates H3K27, which results in the recruitment of PRC1 by the recognition of the histone mark by the Pc chromodomain. However, this simple model is not sufficient to explain PcG silencing. PRC1 and PRC2 components are still visible by immunostaining at many sites on polytene chromosomes in *pho* and *pho*-*like* double mutants [46], suggesting that additional DNA-binding factors are likely to be involved in PcG recruitment and silencing. In addition, competing studies disagree about the extent of colocalization between Pho and PRC1. One puts the percentage at 96% colocalization between Pho and PRC1 (as mapped by the intersection of Pc and Ph), whereas the other reports only 50% between Pho and PRC1 (as mapped by Pc alone) [74,75]. One aspect that may confound comparisons of this nature is that there may be inherent differences between the chromatin from different sources (i.e. tissue culture versus embryo versus different tissues). The antibodies used also play a role, as two separate validated antibodies to the same PcG subunit produce different patterns [76]. Another consideration is that there are many peaks observed for the individual PcG factors that are not shared by other members of the PcG. In addition, the definition of PREs for ChIP studies is based on co-enrichment peaks, most of which have not been functionally validated as PREs. Furthermore, numerous transcription factors have binding sites within each functionally validated PRE (see below).

In reviewing the characterized biochemical activities available to the MSL and PcG complexes, it is clear that both groups have proteins with the ability to interact with DNA or chromatin. Both groups are capable of catalysing histone modifications known to be associated with chromatin state, and both have ubiquitin ligase activity. The general DNA/chromatin affinity observed for these complexes makes logical sense, because this would allow maintenance of chromatin states after their initial establishment. However, for MSL and PcG, many details regarding their initial targeting are still lacking.

## 4. Sequence motifs and binding proteins

In the spreading model, the MSL complex initially binds a set of CESs containing a degenerate sequence motif (figure 2*c*). Reduction of the MSL complex by genetic [2] or RNAi means [77] revealed 150–300 CESs, as defined by perdurance of the ChIP-enriched peaks. The majority contain a (GA)_4_-core sequence, with flanking GA enrichment encompassing a 21–29 bp motif, termed the MSL-recognition element (MRE). CESs moved to autosomes as 150 bp transgenic segments attract the MSL complex to autosomes, whereas mutants in which the consensus MRE is disrupted fail to attract the complex. However, the MRE is enriched only on the X chromosome by 1.5- to 2-fold (depending on stringency of motif search parameters); yet from this modest enrichment, there is nearly perfect fidelity for the X chromosome. Philip *et al.* [78] looked into this conundrum further, analysing the sequence composition biases of the X chromosome versus the autosomes, and MSL-bound versus MSL-unbound genes on the X. Analysis was carried out by parsing the chromosomes and analysing the frequencies of all 2–6-mer ‘words’. They determined that X chromosome genes are characteristically GC rich, not just in *D. melanogaster*, but also in the greater *Drosophila* genus. Thus, an interesting possibility is that primary sequence composition surrounding the MRE motif may play a role in site selection. Credence to this idea is lent by subsequent work which detected a characteristic GC enrichment signature in flanking sequences around MREs [3]. However, additional unknown specificity factors must still be invoked to explain the strong fidelity of the MSL complex for the X chromosome.

To identify additional proteins involved in targeting of the MSL complex, including general factors that might carry essential functions in both sexes, Larschan *et al.* [79] used an RNAi screen that culminated in the identification of a novel zinc-finger protein, CG1832, also linked biochemically to the MSL complex by ChIP-mass spectrometry [80]. In a major step forward, CG1832, renamed chromatin-linked adaptor for MSL proteins (CLAMP), was discovered to have direct sequence-specific DNA-binding affinity for the MRE motif *in vivo* and *in vitro* (figure 2*d*) [4]. However, CLAMP is bound to MRE sequences throughout the genome and in both sexes, so male X specificity is still only partially explained, as follows. MSL complex and CLAMP mutually reinforce each other's interaction at CESs. Furthermore, CLAMP is found at more MREs on the X chromosome than on the autosomes in female cells, suggesting that CLAMP has higher affinity for the X even in the absence of the MSL complex. Based on assessing relative occupancy levels of CLAMP at sites on the X, Soruco and co-workers [4] suggest a model in which binding at the roX2 locus and adjacent chromatin acts as a ‘beacon’, along with the roX RNAs themselves, for MSL recruitment and synergistic spread to MREs along the X. Thus, the characterization of CLAMP provides a direct link between the MSL proteins and MRE sequence-specific recognition, and, along with roX RNAs, provides at least a partial explanation for initial X recruitment.

In contrast to the MSL complex, the structural and functional analysis of PREs, which were originally characterized in the Bithorax cluster, has shown that there is no strong consensus sequence that can be simply defined as a PRE motif. Instead, PREs often contain diverse combinations of sequence motifs for multiple DNA-binding proteins, including Pho/Phol, SP1/KLF proteins, GAGA factor (GAF)/Pipsqueak (Psq), Dorsal switch protein 1 (Dsp1), Grainyhead (Grh) and Zeste [81], reflecting a constellation of transcription factors that may establish initial target gene expression levels (figure 3*b*).

The core consensus motif of Pho is GCCAT, and longer versions of the Pho consensus sequence have been detected by genome-wide analyses [74,75,82]. If we consider PREs as the ChIP-enriched peak of PRC1 (intersection of Pc and Ph), then Pho sites are estimated to overlap with approximately 96% of mapped PREs. The importance of Pho binding motifs in PRE-mediated silencing has been demonstrated by transgenic analyses using diverse PREs [54,83,84]. Pho-like (Phol) has 80% sequence identity with the zinc-finger region of Pho and can also bind to the Pho consensus motif *in vitro*. Furthermore, double mutants of *pho* and *phol* have a synergistic effect leading to Hox gene misexpression, suggesting that Pho and Pho-like act redundantly in Hox gene silencing [46]. However, genome-wide analysis of Phol binding shows far less overlap with Ph sites (21%) compared with the overlap observed between Pho and Ph (see above) [74]. Certainly, Pho and Phol play a major role (perhaps even *the* major role) in PcG targeting, but, because the other complexes can still locate a subset of their sites on chromatin in their absence, clearly there must be other mechanisms of recruitment to such loci. Additionally, Pho is not sufficient for PRE activity, as demonstrated by the large percentage of Pho sites that are not bound by Pc and Ph.

The Sp1/KLF protein consensus sequence is (G/A)(G/A)GG(C/T)G(C/T), and the *engrailed* (*en*) PREs contain a perfect match to this consensus [85]. Spps, one of the SP1/KLF family members, not only binds to the *engrailed* PREs, but also shows an identical binding pattern to Psc on polytene chromosomes. In addition, depletion of Spps leads to a loss of pairing-sensitive silencing (PSS; figure 3*c*), a phenomenon that strengthens PRE-mediated repression of mini-white reporter transgenes in flies homozygous for the transgene [86].

GAGA factor (GAF) and Pipsqueak (Psq) bind to a GAGAG sequence motif. Both GAF and Psq contain a BTB/POZ domain, which is involved in the formation of homo- or heterodimers, and can interact with each other [87]. Genome-wide analysis revealed that GAF is colocalized at about half of Ph binding sites [74]. GAF binding sites in the *even skipped* (*eve*) PRE are necessary for PcG-mediated silencing, and GAF is required for binding of Pho to PRE chromatin *in vitro* [83,88]. Subsequently, GAF binding sites have also been shown to be required in other PREs [81]. GAF is reported to remodel chromatin *in vitro* [89] and recruit chromatin remodelling factors [90], suggesting a role of GAF at PREs is to mediate depletion of nucleosomes to allow binding of other regulators.

The consensus sequence of Dsp1, GAAAA, is found at positions close to or overlapping with Pho sites in diverse PREs. Removal of Dsp1 binding from the *Fab-7* PRE of *abdominal-B* (Abd-B) and one of the *en* PREs results in loss of PcG-mediated silencing [91]. Genome-wide analysis shows that Dsp1, like GAF, is present at about half of Ph binding regions, despite the Dsp1 consensus sequence not showing a strong correlation with the observed Dsp1 genomic localization [74].

The Grh and Zeste consensus sequences are (A/T)C(T/C/A)GGTT and (T/C/G)GAGTG(A/G/C), respectively. Grh binds to the *iab-7* PRE and also interacts with Pho *in vitro* and genetically [92]. Zeste was initially reported as a component of the PRC1 complex [32] and is required for maintenance of Ubx repression in the embryo [93]. However, in genome-wide analysis, Zeste is present only at a small percentage of Ph binding sites (25%) and Pho binding sites (10%) [74,82]. Additionally, *zeste* mutants have no observed mutant phenotype (homeotic or otherwise), suggesting it may be redundant or non-essential [94].

Paradoxically, many of these DNA-binding proteins with consensus motifs found in PREs are also implicated in transcriptional activation, and, furthermore, cognate mutants do not show clear PcG mutant phenotypes [95]. In fact, an entire group of transcriptional activators, the Trithorax group (TrxG), are also found to localize to many PREs. The TrxG is typically thought of as antagonistic to PcG function, and models propose that competition between the TrxG and PcG for binding at PREs may play a role in maintenance of transcriptional state [96]. An algorithm using the consensus motifs discussed above to predict PRE sites identified 167 candidates [97], but only 32 sites overlapped with Ph binding sites revealed by genome-wide analysis [74]. The low predictive power of this algorithm shows that there are likely to be many other factors and parameters for PRE recognition that are still unknown.

## 5. Local chromatin environment

**Figure 4.**
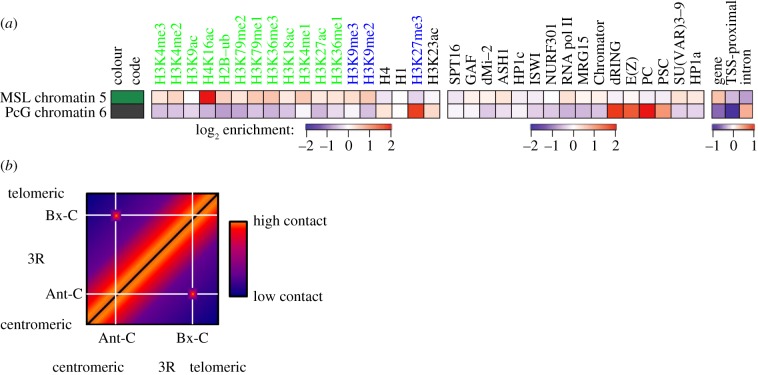
Chromatin environment and long-range chromatin interactions. (*a*) Analysis of 18 histone modifications (left) by the modEncode project led to the identification of signatures for nine different chromatin types. Type 5 (green) bore the signature of dosage compensated chromatin (note the enrichment of the MOF-catalysed H4K16Ac mark), whereas type 6 (grey) was identified as PcG-associated chromatin (note the enrichment of the E(z) catalysed H3K27me3 mark). Cross-validation with other chromatin proteins and with genomic features (right) confirms many of the predicted enrichments/depletions. From Kharchenko *et al.* [98]. (*b*) Schematic for results of Hi-C experiments that capture long-range interactions between PcG-bound regions. The highest-frequency contacts are observed along the diagonal owing to the fact that interactions are strongest over short linear distances. However, high contact frequencies are observed between the Bithorax and Antennapedia clusters, which are regulated by the PcG. Adapted from Sexton *et al.* [99].

The modENCODE project has catalogued the distribution of selected chromatin marks in the *Drosophila* genome. The aggregate data for 18 histone modifications have revealed nine major chromatin types based on their combinatorial composition. Cross-analysis also included binding of a number of non-histone chromatin proteins and DNaseI hypersensitivity, as well as gene structure and expression to further describe the state of the chromatin. Unsurprisingly, transcriptionally active chromatin and repressed chromatin were easily identifiable. Different states of transcription (i.e. transcriptional start sites, transcriptional elongation) and different types of repressed chromatin (i.e. pericentromeric heterochromatin, PcG-repressed domains) each bore their own unique signatures. The male X chromosome displayed a unique form of active chromatin, characterized by the H4K16Ac mark, a hallmark of MSL-mediated dosage compensation (figure 4*a*) [98].

The modEncode data allowed Alekseyenko *et al.* [3] to explore the role of local chromatin environment in the choice of MRE sequences to be used as CESs. They compared the chromatin environment of 150 bound CESs with several alternative groups of unused MREs. The results showed that a clear signature for functional MREs consists of an enrichment of the H3K36me3 mark, Jil-1 (a kinase enriched on X with the MSL complex) and, unsurprisingly, the H4K16Ac mark. Notably, the enrichment of these three factors is present in both male and female tissue culture cells, suggesting that they are more than merely a post-MSL binding consequence. Furthermore, as mentioned above, a characteristic sequence bias exists around functional MREs. The surrounding 1 kb (centred on the MRE) showed an AT enrichment, whereas the surrounding 10 kb (excluding the central 1 kb) was characteristically GC rich. Building a predictive model using chromatin marks and the AT richness as factors met with reasonable success, suggesting that chromatin environment is likely to play a role in initial targeting of the MSL complex. However, even given enrichment for all of these the factors, it remains impossible to discriminate with certainty between a functional MRE and one that is not used. The ultimate puzzle to be solved is why autosomal sites meeting all parameters still do not autonomously attract the MSL complex, whereas similar-looking sequences moved from X to A do function to attract the complex.

PREs have different combinations of diverse motifs without any preferential number, order or spacing [54]. Furthermore, the number and order of motifs show significant differences among *Drosophila* species, even within orthologous PREs [54]. It is unclear, however, whether the diverse PRE topologies have different functional effects or whether they have similar functional activities independent from their sequence organization. To investigate this question, selected PREs have been analysed using transgenes that contain a PRE and reporter gene, such as mini-white or lacZ; these transgenic analyses have revealed that the activities of PREs are highly influenced by genomic context. For example, transgenes containing *en* or *invected* (*inv*) PREs show PSS at only about 60% and 20–45% of insertion sites, respectively [100]. This phenomenon may be due to the effects of neighbouring regulatory elements on PRE activity. In screening dominant suppressors of PSS, Noyes *et al*. [101] observed that gain of function mutations in the transcriptional activator Woc can block *en* PRE activity. This suggests that, in the context of this reporter assay, there is competition between the PRE and neighbouring regulatory elements, such as enhancers, for control over the transcriptional state of the reporter gene.

Insulators have been identified as another type of regulatory element that affects PRE activity, potentially by blocking the spreading of PcG proteins or H3K27me3 marks [102–104]. Genome-wide analysis shows that diverse insulator proteins such as Su(Hw), CP190 and dCTCF are broadly distributed throughout the genome [105,106]. Interestingly, PREs at many sites, including the Hox gene region, are flanked by insulator elements, suggesting that the flanking insulators protect neighbouring genes from inappropriate silencing by PREs as well as inappropriate activation by enhancers. The removal of insulator binding sites or the depletion of insulator proteins can result in lower H3K27me3 within these domains, but appears to have little effect on spreading beyond the borders, which might be expected if insulators were the sole causative agent [107]. Alternatively, a recent study using a *Fab*-7 PRE transgene showed that spreading of H3K27me3 is blocked by RNA polymerase II bound promoter regions and active chromatin marks rather than by insulator elements [40,108,109]. It is likely that multiple mechanisms contribute to the delineation of a PcG domain.

The sensitivity of PREs to genomic context makes comparison of functional differences between PREs challenging. Therefore, site-specific recombination tools such as gene conversion and ΦC31 integration have been very important to examine the effects of mutation or deletion of sites within a PRE, and for direct comparison between different PREs in a constant genomic context [84,110]. Using the gene conversion technique, Kozma *et al.* [84] showed that binding motifs of Pho and GAF in the *bithoraxoid* (*bxd*) PRE are cooperatively required for silencing by the PRE, whereas the Dsp1 binding motif is not essential for the PRE activity. In addition, replacement of the *bxd* PRE in a reporter construct with the *iab-7* or *iab-5* PREs from *Abd-B* revealed that these PREs are interchangeable, indicating equivalent functional capabilities. On the other hand, direct comparison between the *Fab-7* PRE and one of the *vestigial* (*vg*) PREs at four ΦC31 site-specific integration loci demonstrated that these two PREs exhibit differential silencing traits in a genomic-context-dependent manner, indicating that different PREs can have distinct properties [110]. Furthermore, a recent functional analysis of two different *en* PREs supports the idea that different PREs produce different functional outcomes. PRE1 and PRE2 of the *engrailed* gene not only have a different number, order and spacing of motifs, but also require different numbers of Pho motifs for silencing activity. In addition, an AT-rich region only found in PRE1 is required for full PSS activity. These differences led to distinct expression patterns in embryonic and larval reporter assays in which the reporter construct was integrated at the same genomic site [54].

## 6. Higher-order chromatin organization

An additional avenue that has yet to be fully explored with respect to MSL complex targeting is the three-dimensional structure and folding of the genome [111]. This piece of the puzzle regarding the larger chromatin environment has been the subject of intense investigation by the chromatin field recently [99,112], but without specific attention to dosage compensation. The X chromosome, like all chromosomes, clearly forms its own territory within the nucleus. However, small 150 bp CES segments inserted on autosomes can still attract the MSL complex to their ectopic sites, so any model invoking global chromosomal structure will need to incorporate the apparent ability of these segments to act autonomously wherever inserted.

Much more intensive investigation of higher-order structure has occurred in the PcG field. Through PRE transgenic analyses and techniques such as DNA adenine methyltransferase identification (DamID), chromatin conformation capture assays (3C and associated technologies), and fluorescent *in situ* hybridization, it has been reported that PcG-targeted regions can contact other PcG-bound regions or promoters over long distances [113–116]. On the basis of these observations, it has been proposed that the long-distance contacts are mediated by interaction between PcG proteins bound at distant PREs, creating large loop structures that contribute to higher-order chromatin structure. Such interactions clearly exist between the BX-C and ANT-C, as observed by recent Hi-C chromatin capture experiments (figure 4*b*) [99]. In further support of this idea, PcG proteins are visualized by immunofluorescence as nuclear speckles termed ‘Polycomb bodies’, of which there are significantly fewer than the number of PcG binding sites found by genome-wide analysis. Therefore, Polycomb bodies are thought to be foci formed by long-range interactions among PcG proteins bound to several PREs, even if large PcG binding chromatin domains spanning more than 100 kb (such as Hox gene cluster regions) may by themselves be visualized as Polycomb bodies.

**Figure 5.**
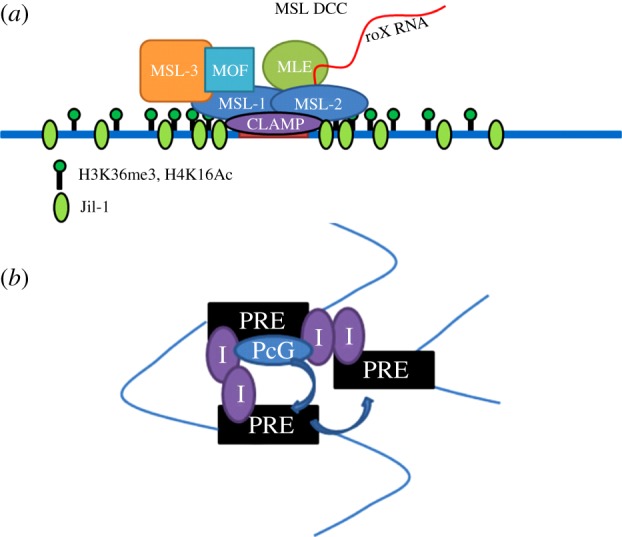
Updated models. (*a*) The core MSL complex is targeted to MREs in CESs in part by the DNA-binding activity of CLAMP, which provides a molecular link between the complex and DNA in a sequence-specific manner. Selection of CESs also strongly favours an active chromatin context, with pre-existing enrichment of the histone marks H3K36me3 and H4K16Ac and the chromosomal kinase Jil-1 that can be observed even in female cells (thus, not due to the presence of the MSL complex itself). (*b*) Insulators contribute to the long-range interactions between PREs. In this way, PcG complexes may be brought to distal sites of activity. As yet unresolved, however, is the identity of factors that are critical for initial targeting of the PcG complexes. Certainly, Pho plays a major role, but other, unidentified factors also clearly take part.

Recently, however, the idea that PcG proteins themselves mediate long-range interaction has been challenged. Using transgenes containing the *Mcp* and *Fab* elements, Li *et al.* [117] showed that the interaction between *Mcp* and *Fab-7* elements does not depend on the ability to recruit PcG complexes, but rather on insulator DNA sequences flanking or within the PRE element. In subsequent investigations by the same group, CTCF binding sites were observed to be critical for long-range interactions of reporter constructs; in comparison, the PRE was observed to be needed only for proper and stable sorting to subnuclear structures [118]. Additionally, this work suggests that interactions between insulators, enhancers and PREs may be quite complex; perhaps so much so that such interactions may, in reality, be difficult to model with ectopic transgenic methodology and individual transgenes (figure 5).

## 7. Involvement of RNAs

In review of the topics covered so far—the biochemical properties of the proteins and complexes themselves, the sequence motifs associated with their binding, and the state of the chromatin around targets, both locally and at longer range—it seems clear that each of these factors plays at least an incremental role in determining the initial targeting of chromatin complexes. In this section, we consider evidence for the role of RNAs in the initial targeting of these model complexes.

The long non-coding roX RNAs are an integral part of the MSL complex [119]. They are critical for the establishment of the full pattern of MSL binding along the male X chromosome [120]. However, in the absence of both transcripts, there is still limited assembly and targeting of the complex. In a recent advance, biochemical analysis provided evidence that ATP-dependent remodelling of the stem-loop structures of the roX RNAs by MLE is a critical step in complex assembly [58]. Interestingly, mutation of the structure of these loops can lead to mislocalization of the MSL complex [121,122]. However, in all the cases of roX mutation, residual targeting of the X remains intact, suggesting that the RNA may function primarily in the spreading rather than the initial targeting step. Long non-coding (lnc) RNAs have not been reported in PcG targeting in *Drosophila*, in contrast to reports in mammalian systems, in which numerous lncRNAs have been proposed to function in PcG targeting [123].

A recent report proposes a role for the small interfering RNA (siRNA) pathway in targeting of the MSL complex to the X chromosome. Menon & Meller [124] observe a synthetic lethal phenotype in male flies between roX RNA double mutants and mutants for the siRNA pathway such as Dicer-2 (Dcr-2), Argonaute 2 (Ago2), and Elongator complex protein 1 (D-elp1). The synthetic lethal phenotype is likely to be due to an accompanying defect in X-chromosome-specific MSL recruitment. The aberrant targeting in these cases ranged from lack of X recruitment, to relocalization to the chromocentre, to appearance at autosomal or telomeric sites. The involvement of ncRNAs other than the roX RNAs in dosage compensation would be a potentially rich source of additional targeting factors. Menon and Meller note, however, that it is possible that the observed phenotypes may not come from a direct interaction between the siRNA machinery and the MSL complex at dosage compensated loci, but rather from a more general role of the siRNA machinery in chromatin architecture.

Similarly, the direct involvement of RNAi machinery components in PcG-mediated silencing is being debated in the PcG field. The positive correlation of Ago2 ChIP data with PcG ChIP-chip data from modENCODE led Taliaferro and co-workers to suggest that Ago2-mediated transcriptional repression, which is independent of the catalytic activity of Ago2, might be related to PcG-mediated silencing [125]. Previously, Grimaud and co-workers [126] found that mutations in RNAi components such as Ago1, Piwi, Aubergine (Aub) and Dcr2 resulted in the derepression of PcG-mediated silencing in PSS assays. However, the appearance of *Fab-*7 region directed 21–23 nt small RNAs in transgenic flies was due solely to the *Fab-7* element in the transgene; the endogenous locus itself does not stimulate the production of such siRNAs.

Therefore, the role of RNAi components in PcG-mediated silencing still seems uncertain, and potentially tied to the structure of the specific transgenes used in transgenic assays. A recent analysis of the possible role of RNAi components in PcG-mediated silencing at the endogenous BX-C locus showed that depletion of RNAi components did not result in any significant change in the repressed chromatin state of the locus, and direct comparison of Ago2-bound small RNAs and PcG-target promoter regions showed no enrichment of small RNAs for such PcG targets [127]. In addition, Moshkovich *et al*. showed that the binding profile of Ago2 overlaps with TrxG proteins as well as PcG proteins, and Ago2 not only preferentially associates with active promoters, but also acted as a TrxG protein opposing the *Pc* mutant phenotype in a genetic screen [128].

In one additional potential link to small RNAs, analysis of the chromatin environment at transcription start sites proximal to PREs showed that these regions are enriched with H3K4me1 and H3K4me2 marks, and many of them generate short RNAs owing to stalled PolII [98], consistent with the possibility that 5’ paused RNAs might play a functional role in PcG targeting [129].

## 8. Summary

As can be seen, the constellation of factors and sequence characteristics that may be involved in initial targeting of complexes to chromatin makes investigation of this topic a complicated endeavour. In the case of MSL targeting, primary sequence characteristics of CESs are certainly a major factor, as novel sex chromosomes generated by chromosomal fusions in *Drosophila miranda* become dosage-compensated by the acquisition of CES-like sequences [130]. The recent characterization of CLAMP has provided a strong molecular link between the MSL complex and its recruitment to MREs in CESs. Whether the *D. miranda* version of CLAMP can similarly explain the selection of CES-like sequences will be an important test. Interestingly, although the mechanisms of dosage compensation are not conserved in nematodes and in mammals, two other model systems that have been intensively studied, the ‘nucleate and spread’ models for targeting to the X chromosome, appear quite similar in the three systems [51,131,132].

PcG targeting seems to involve multiple proteins, and no single consensus sequence (even the Pho/Phol consensus) can adequately predict PcG binding. Furthermore, the manner by which PREs are typically studied is with transgenic reporter assays in which recruitment of PcG is not directly assayed; rather, functional silencing of the transgene is tested. However, PcG recruitment does not always lead to silencing, but may instead only modulate transcription levels (e.g. at the Psc/Su(z)2 locus) [104,133–135]. PcG targeting and silencing function, then, may not always be linked. The molecular details of this separation are unclear; however, post-translational modifications of PcG subunits, in addition to the diverse biochemical activities of the multiple complexes, suggest that there could be factors that influence one but not the other [49,136,137].

With the advent of higher-throughput genome editing technologies, such as the CRISPR/Cas9 system, it is becoming more feasible to do the types of experiments that may reveal requirements for targeting and function without removing sites from their endogenous locations. It may finally be possible to expand from single site assays to look at more global recruitment: mutation of a majority of MREs or PREs in a chromosomal region. New chromatin immunoprecipitation techniques that enhance the resolution of binding sites may also help to refine known binding patterns or reveal sequence motifs. Hopefully, these technologies will allow investigators to address the many outstanding questions that still exist in the area of PcG and MSL initial targeting.
